# Complete mitochondrial genome and the phylogenetic position of the *Mugilogobius myxodermus*: an endemic freshwater gobiid fish in China

**DOI:** 10.1080/23802359.2017.1372708

**Published:** 2017-09-04

**Authors:** Lei Cai, Ren Huang, Jianjun Li

**Affiliations:** Key Laboratory of Guangdong Laboratory Animals, Guangdong Laboratory Animals Monitoring Institute, Guangzhou, Guangdong, China

**Keywords:** Mitogenome, *Mugilogobius myxodermus*, Gobiidae, phylogeny

## Abstract

The *Mugilogobius myxodermus* is an endemic freshwater fish in china. Herein, we sequenced and assembled the first complete mitogenome of *M. myxodermus.* The total length of the mitogenome is 16,495 bp, containing 13 protein-coding genes, two ribosomal RNAs (rRNA), two non-coding regions (control region and origin of light-strand replication), and 22 transfer RNAs (tRNA). The overall base composition of *M. myxodermus* is 26.76% T, 28.97% C, 27.81% A, and 16.45% G, respectively. A phylogenetic tree showed that *M. myxodermus* clustered closest to *M. abei.* The complete mitogenome of *M. myxodermus* provides a resource for studies on biogeography and evolution of this gobiid fish.

The *Mugilogobius myxodermus* (Herre 1935) is an endemic freshwater fish in south china, distributed mainly in Yangtze River, Ou River, Jiulong River, and Pearl River system (Wu and Zhong 2008). Evolutionarily and ecologically, *M. myxodermus* is the only freshwater demersal fish of genus *Mugilogobius* in China (Wu and Zhong 2008). Meanwhile, it can also have potential to be an ornamental fish.

In this study, the complete mitogenome of *M. myxodermus* was sequenced (GenBank accession No. MF537252). The specimens of *M. myxodermus* were collected from the Lake Tai at Wuxi, China (geospatial coordinates: 31°30 N, 120°07 E) and identified by morphology (Larson 2001). One specimen (Voucher no. 3144C0001000000008) was stored in 100% ethanol at National Infrastructure of Laboratory animal Resources (http://www.lasdr.cn/pages/resdata_dataview.jsp?id=10069100). Total genomic DNA was extracted from the tail fin with the HiPure Tissue DNA Mini Kit (UMagen, Guangzhou, China). The versatile primers were designed on the basis of the mitogenome sequence of *Mugilogobius abei* (GenBank Accession No. NC_023353) (Huang et al. 2016). The PCR-based mitogenome sequencing was conducted in accordance to what was reported previously (Miya and Nishida 1999; Cai et al. 2016). The final assembly was annotated using the MitoAnnotator (Iwasaki et al. 2013).

The complete mitogenome of *M. myxodermus* is 16,495 bp, containing 13 protein-coding genes, two ribosomal RNAs (rRNA), two non-coding regions (control region and origin of light-strand replication), and 22 transfer RNAs (tRNA). Except for the eight *tRNAs* (*tRNA^Gln^*, *tRNA^Ala^*, *tRNA^Asn^*, *tRNA^Cys^*, *tRNA^Tyr^*, *tRNA^Ser1^*, *tRNA^Glu^*, and *tRNA^Pro^*) and *ND6* genes encoded on the L-strand, all other mitochondrial genes are encoded on the H-strand. The overall nucleotide composition of *M. myxodermus* is 26.76% T, 28.97% C, 27.81% A, and 16.45% G, respectively. All 13 protein-coding genes start with ATG except *COXI* which starts with GTG. Most of the protein-coding genes (10 of 13 genes) end with TAA, TAG, and AGG, the other three genes (*COXII*, *COXIII*, and *CYTB*) have T or TA incomplete stop condon, which are very typical in many other gobiid fishes (Chen and Wen 2016; Zhang et al. 2016; Huang et al. 2017). *M. myxodermus* contains two non-coding regions: the control region (D-loop) is 850 bp in length, which is located between *tRNA^Pro^* and *tRNA^Phe^* genes; another small non-coding region, which is a 36 bp fragment and the origin of light-strand replication (O_L_), is located between the *tRNA^Asn^*and *tRNA^Cys^*genes. The gene content, structure, and arrangement of mitogenome in *M. myxodermus* are similar to those observed in other fishes (Behera et al. 2017; Mochizuki et al. 2017; Ruck et al. 2017; Ulmo-Díaz et al. 2017).

All available full mitogenome sequences of subfamily Gobionellinae and two non-percomorph outgroups (*Danio rerio* and *Oryzias latipes*) were mined from GenBank. A maximum likelihood phylogenetic analysis was conducted using the software Mega 6 (Figure 1) (Tamura et al. 2013). *M. myxodermus* was phylogenetically placed near the *M. abei* and *M. chulae*, with a very short branch length. The complete mitochondrial genome of *M. myxodermus* will be useful for further evolution and biogeography studies of gobiid fishes.

**Figure 1.**
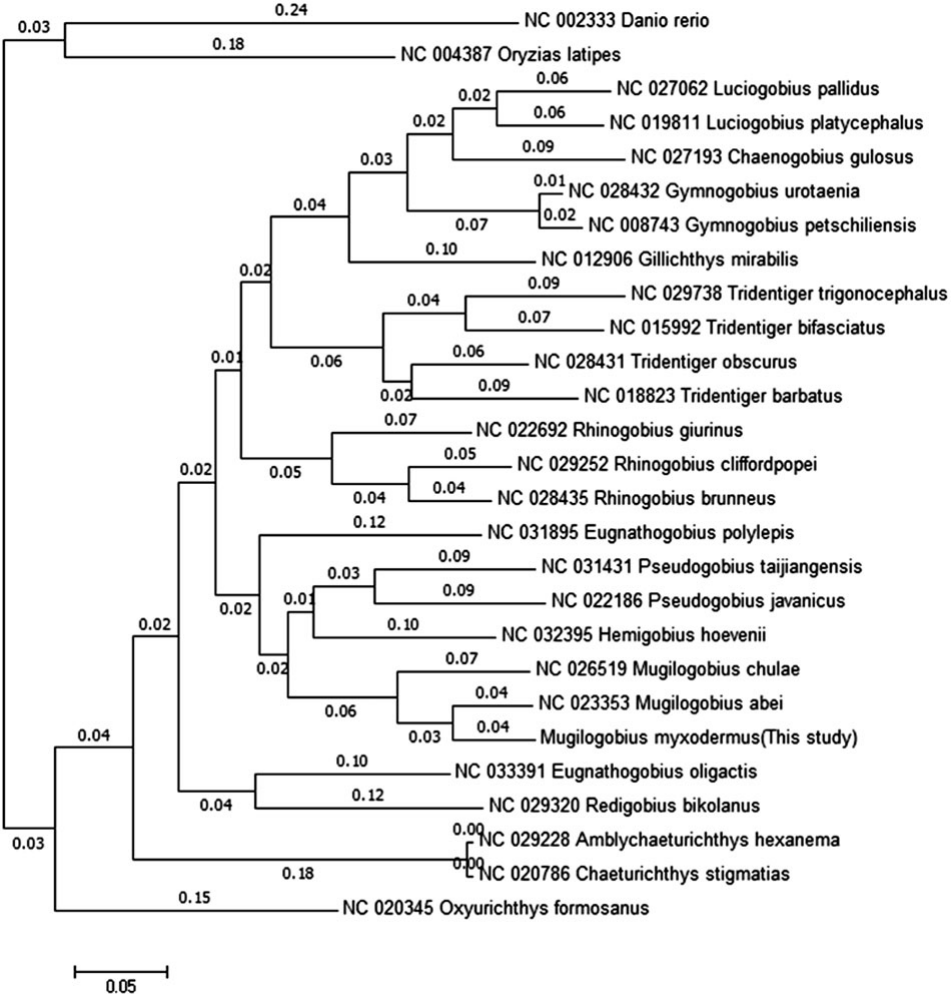
A maximum likelihood molecular phylogenetic tree of subfamily Gobionellinae and two non-percomorph outgroups. Values on nodes are branch lengths. The GenBank accession numbers are indicated before the name.
